# Development and Validation of a Tool Integrating the 21-Gene Recurrence Score and Clinical-Pathological Features to Individualize Prognosis and Prediction of Chemotherapy Benefit in Early Breast Cancer

**DOI:** 10.1200/JCO.20.03007

**Published:** 2020-12-11

**Authors:** Joseph A. Sparano, Michael R. Crager, Gong Tang, Robert J. Gray, Salomon M. Stemmer, Steven Shak

**Affiliations:** ^1^ Montefiore Medical Center, Albert Einstein College of Medicine, Bronx, New York; ^2^ Exact Sciences, Redwood City, CA; ^3^ University of Pittsburgh, NRG Oncology Statistical and Data Management Center, Pittsburgh, PA; ^4^ Dana Farber Cancer Institute, ECOG-ACRIN Statistical Center, Boston, MA; ^5^ Davidoff Center, Rabin Medical Center, Petah Tikva, Israel; Sackler Faculty of Medicine, Tel Aviv University, Tel Aviv, Israel

## Abstract

The 21-gene recurrence score (RS) is prognostic for distant recurrence (DR) and predictive for chemotherapy benefit in early breast cancer, whereas clinical-pathological factors are only prognostic. Integration of genomic and clinical features offers the potential to guide adjuvant chemotherapy use with greater precision.

**METHODS:**

We developed a new tool (RSClin) that integrates RS with tumor grade, tumor size, and age using a patient-specific meta-analysis including 10,004 women with hormone receptor–positive, human epidermal growth factor receptor 2–negative, and node-negative breast cancer who received endocrine therapy alone in the B-14 (n = 577) and TAILORx (n = 4,854) trials or plus chemotherapy in TAILORx (n = 4,573). Cox models for RSClin were compared with RS alone and clinical-pathological features alone using likelihood ratio tests. RSClin estimates of DR used a baseline risk with TAILORx event rates to reflect current medical practice. A patient-specific estimator of absolute chemotherapy benefit was computed using individualized relative chemotherapy effect from the randomized TAILORx and B-20 trials. External validation of risk estimation was performed by comparing RSClin estimated risk and observed risk in 1,098 women in the Clalit registry.

**RESULTS:**

RSClin provides more prognostic information (likelihood ratio χ^2^) for DR than RS or clinical-pathological factors alone (both *P* < .001, likelihood ratio test). In external validation, the RSClin risk estimate was prognostic for DR risk in the Clalit registry (*P* < .001) and the estimated risk closely approximated the observed 10-year risk (Lin concordance 0.962). The absolute chemotherapy benefit estimate ranges from 0% to 15% as the RS ranges from 11 to 50 using RSClin in a 55-year-old woman with a 1.5-cm intermediate-grade tumor.

**CONCLUSION:**

The RSClin tool integrates clinical-pathological and genomic risk to guide adjuvant chemotherapy in node-negative breast cancer and provides more individualized information than clinical-pathological or genomic data alone.

## INTRODUCTION

The 21-gene recurrence score (RS) assay provides both prognostic information for distant recurrence (DR) and predictive information for chemotherapy benefit in hormone receptor–positive, human epidermal growth factor receptor 2 (HER2)–negative early breast cancer.^1-5^ The relative risk reduction with chemotherapy increases with increasing RS result, and the absolute magnitude of chemotherapy benefit also varies with the underlying recurrence risk,^6^ which is associated with both clinical-pathological features and RS result.^7^

**CONTEXT**

**Key Objective**
Our goal was to develop a tool that integrates clinical-pathological and genomic information in early breast cancer.
**Knowledge Generated**
Using a patient-specific meta-analysis, we developed a new tool (RSClin) that integrates the 21-gene recurrence score with tumor grade, tumor size, and age to provide estimates of 10-year distant recurrence (DR) rates and absolute chemotherapy benefit.
  We demonstrated that RSClin provides more prognostic information for DR than recurrence score or clinical-pathological factors alone and that RSClin estimates closely approximately actual DR risk in an independent real-world data set.
**Relevance**
The new RSClin tool provides individualized information for DR risk and chemotherapy benefit that may be used to guide the use of adjuvant chemotherapy in hormone receptor–positive, human epidermal growth factor receptor 2–negative, node-negative breast cancer.

The RS-pathology-clinical (RSPC) tool, which integrates prognostic information for DR from the RS result with tumor grade, tumor size, and patient age, is more prognostic for DR than RS result alone in a patient-specific meta-analysis (PSMA) of 1,735 women with hormone receptor–positive early breast cancer treated with adjuvant endocrine therapy in the NSABP B-14 trial (between 1982 and 1988) and TransATAC trial (between 1996 and 2000), of whom 10% had HER2-positive breast cancer.^8^

Here, we report the development and validation of a new tool for node-negative breast cancer providing prognostic estimates of DR risk with endocrine therapy alone in HER2-negative breast cancer derived from a larger and more contemporaneously treated cohort than that used for the development of RSPC and adding estimation of incremental absolute chemotherapy benefit not previously provided by RSPC. The tool was derived from a PSMA in 10,004 women with hormone receptor–positive, HER2-negative, and node-negative breast cancer, of whom 9,427 participated in the TAILORx trial. The TAILORx trial included patients treated between 2006 and 2010, thus more accurately reflecting improved prognosis for hormone receptor–positive breast cancer after the year 2000.^9^ We also report external validation of this new tool for estimation of DR risk in an independent cohort of 1,098 women with node-negative disease from the Clalit Health Services registry.^10^

## METHODS

The prespecified criteria for patient inclusion in the PSMA—model covariates, end point, statistical methods for the prognostic tool (using data from the B-14^1^ and TAILORx^5^ trials with adjustment using B-20^3^), the chemotherapy effect prediction tool (using data from TAILORx^5^ and B-20^3^), and external validation of the prognostic tool (using data from the Clalit Health Services registry^10^)—are described in detail in Table S1 of the Data Supplement (online only) and depicted in the Consort diagram (Data Supplement Fig S1). Human subject protection approval was obtained from the local institutional review boards as described for each individual trial report.^1,3,5,10^

Cox regression models were fit separately to each study with covariates RS result, histologic tumor grade, tumor size, and patient age at surgery. RS result and age in years were fit using two df natural cubic splines with knots at the minimum, median, and maximum values for each study (B-14, TAILORx Arms A and B, and TAILORx Arms C and D). Tumor size in centimeters was fit as a linear covariate. Grade was assessed by a single central laboratory breast cancer pathologist in B-14 and locally in TAILORx. The prespecified end point was time to first DR. DR risk estimation used PSMA methodology,^11^ calculating inverse-variance weighted averages of log cumulative hazard estimates from the individual study on Cox regressions with baselines computed using the event rate of TAILORx arms A or B. Arm A of TAILORx included patients with RS 0-10 assigned to endocrine therapy alone, and arm B included patients with an RS result of 11-25 randomly assigned to endocrine therapy alone. TAILORx arms C and D were included in the PSMA adjusting the patient-specific log cumulative hazard by subtracting the patient-specific log hazard ratio of chemotherapy from B-20 (see the Data Supplement). Arm C included patients with the RS of 11-25 randomly assigned to chemoendocrine therapy, and Arm D included patients with an RS of 26-100 assigned to chemoendocrine therapy.^12^ Weights for TAILORx arms A and B (RS 0-25) and TAILORx arms C and D (RS 11-100) were set to 0 outside the studied RS ranges to avoid extrapolation, within linear transitions to zero weighting over five unit ranges of the RS result. Examples of PSMA weighting of individual studies and intermediate calculations are described in detail below and the Data Supplement.

Patient-specific absolute benefit of chemotherapy was estimated by combining the PSMA risk estimates with a PSMA estimate of relative chemotherapy benefit using B-20 and TAILORx. We first estimated the patient-specific chemotherapy effect log hazard ratio using a multivariate Cox regression of the patients with HER2-negative, hormone receptor–positive breast cancer in B-20, in which women with estrogen receptor–positive breast cancer were randomly assigned to 5 years of tamoxifen or tamoxifen plus adjuvant chemotherapy. A TAILORx patient-specific chemotherapy effect log hazard ratio was estimated as the difference in the patient-specific prognostic model log cumulative hazard estimates for TAILORx arms C and D versus A and B. The B-20 and TAILORx estimates were combined with inverse variance weighting. This PSMA estimate was added to the prognostic model log cumulative hazard estimate under endocrine therapy to estimate the log cumulative hazard with chemoendocrine therapy. Transforming to the risk scale, the patient-specific absolute chemotherapy benefit was calculated as the difference in the risk estimates for endocrine therapy alone and chemoendocrine therapy. A CI was calculated using the delta method (see the Data Supplement).

Model estimates may be calculated for specified endocrine therapy with tamoxifen or aromatase inhibitors using the treatment effect hazard ratio from an Early Breast Cancer Trialists’ Collaborative Group meta-analysis (see the Data Supplement).^13^

A new online tool (RSClin) that provides the estimated 10-year DR risk and absolute benefit assessment is available for use by healthcare professionals.^14^

## RESULTS

### Assessing Original RSPC for DR in TAILORx

Both the old RSPC tool and the new RSClin tool integrate variables including RS, age, tumor size, and tumor grade along with the type of endocrine therapy (tamoxifen or aromatase inhibitor). In a univariate Cox regression, the original RSPC risk estimate, transformed to the log-cumulative hazard scale, was strongly associated with the observed DR in TAILORx arms A and B with an RS result of 0-25 treated with endocrine therapy alone (standardized hazard ratio 1.86; 95% CI, 1.61 to 2.15; *P* < .001). The TAILORx arms A and B study population was divided into quintiles using the original RSPC risk estimates. Within each quintile, the observed 10-year DR risk was calculated from the TAILORx data, assuming that the hazard during 9-10 years equals the hazard during 8-9 years using martingale extension.^11^ The observed risk was plotted against the average RSPC-estimated risk (Data Supplement Fig S2). The estimated risk closely follows the observed risk, but the original RSPC risk estimates were higher than the observed risk estimates in TAILORx, which is consistent with reports indicating improved prognosis for hormone receptor–positive breast cancer after the year 2000.^9^

### Multivariable Cox Regression Analysis for DR

Results of the multivariate Cox regression analyses for B-14, TAILORx arms A and B, and TAILORx arms C and D are shown in Table 1. The hazard ratios for RS result, tumor grade, and tumor size are broadly consistent across studies; the hazard ratio for age shows more variation. The greater hazard ratio for RS result in TAILORx arms A and B (assigned to endocrine therapy alone) relative to TAILORx arms C and D (assigned to chemoendocrine therapy) is consistent with the prediction of chemotherapy effect by RS result observed in the HER2-negative population of the B-20 trial.^3^ The multivariate Cox regression analysis of B-20 used to adjust the TAILORx arms C and D estimates for the effect of chemotherapy is shown in Table S2 of the Data Supplement. The two-way interaction hazard ratio for RS with chemotherapy for the overall population is shown at the bottom of Table S2 in the Data Supplement. It is consistent with the RS and chemotherapy interaction previously reported for patients with HER2-negative breast cancer.^3^

**TABLE 1.**
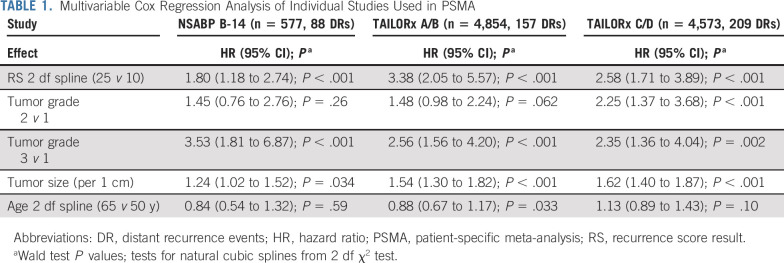
Multivariable Cox Regression Analysis of Individual Studies Used in PSMA

The amount of information for DR prognosis provided by the RSClin model was quantified using the likelihood ratio χ^2^ and compared with the reduced model with RS result alone and with the reduced model with clinical-pathological features alone via likelihood ratio tests. Test results demonstrate that the RSClin model provides significantly more information than the model with tumor grade, tumor size, and age (*P* < .001) and the model with RS result alone (*P* < .001) (Data Supplement Table S3).

### RSClin DR Risk Estimates for Endocrine Therapy Alone

**FIG 1.**
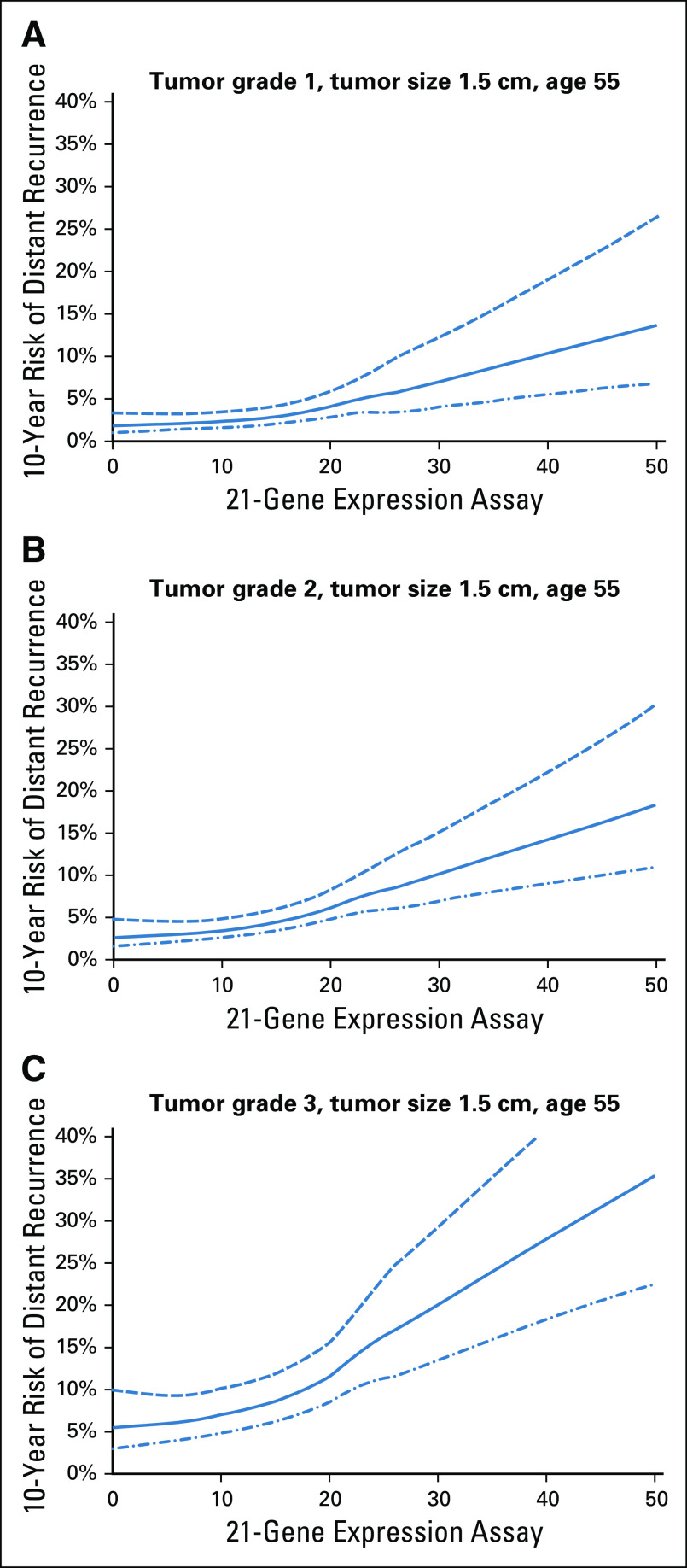
The RSClin tool provides individualized prognosis estimates based on entry of patient information for the RS result, age, tumor size, and tumor grade. Example estimates (solid line) and 95% CIs (dotted lines) provided by the RSClin tool for 10-year distant recurrence risk for endocrine therapy alone with 21-gene expression assay ranging from 0 to 50 for a 55-year-old woman with a tumor size of 1.5 cm, a typical clinical scenario in which the 21-gene RS is commonly used in clinical practice. (A) Results for tumor grade 1; (B) results for tumor grade 2; (C) results for tumor grade 3. RS, recurrence score.

Example estimates of 10-year DR risk with 95% CIs with endocrine therapy alone provided by the RSClin tool for 55-year-old patients with 1.5-cm tumors of low, intermediate, and high grades are shown in Figure 1. Examples with varying tumor size and patient age are shown in Figures S7 and S8 of the Data Supplement. As expected, the risk estimates increase with increasing RS result, tumor grade, and tumor size, and the CIs are larger with high RS results, which occur generally less often.

A scatter plot comparing DR risk estimates with endocrine therapy alone from the original RSPC tool and the RSClin tool using the covariate distribution in TAILORx (n = 9,427) is shown in Figure S9 of the Data Supplement. The median ratio (RSClin tool:original RSPC) is 73.5% (10^th^-90^th^ percentile 66.8%-80.7%). A calibration plot of the observed 10-year DR risk (martingale extension estimates) from TAILORx arms A and B against the average risk estimate provided by the RSClin tool is shown in Figure S10 of the Data Supplement. The observed risk closely follows the estimated risk, suggesting that the functional form of the covariates in the new model and the PSMA studies provides representative estimates.

The DR risk estimates shown here reflect the endocrine therapy use in TAILORx, which was primarily tamoxifen for younger patients and aromatase inhibitors for older patients. Estimates for specified endocrine therapy are implemented in the RSClin online tool.

### RSClin Individualized Estimates of Absolute Chemotherapy Benefit

Patient-specific absolute chemotherapy benefit is estimated by combining relative treatment effect estimates from a PSMA of B-20 and TAILORx with the new prognostic tool DR risk estimates. Since the endocrine therapy arm of B-20 was used in the development of the 21-gene RS assay, bias in the B-20 estimate of its relationship with chemotherapy effect cannot be ruled out. However, the prospective TAILORx study gives very similar estimates of chemotherapy effect versus RS result (Data Supplement Fig S3), suggesting that the bias is not substantial.

**FIG 2.**
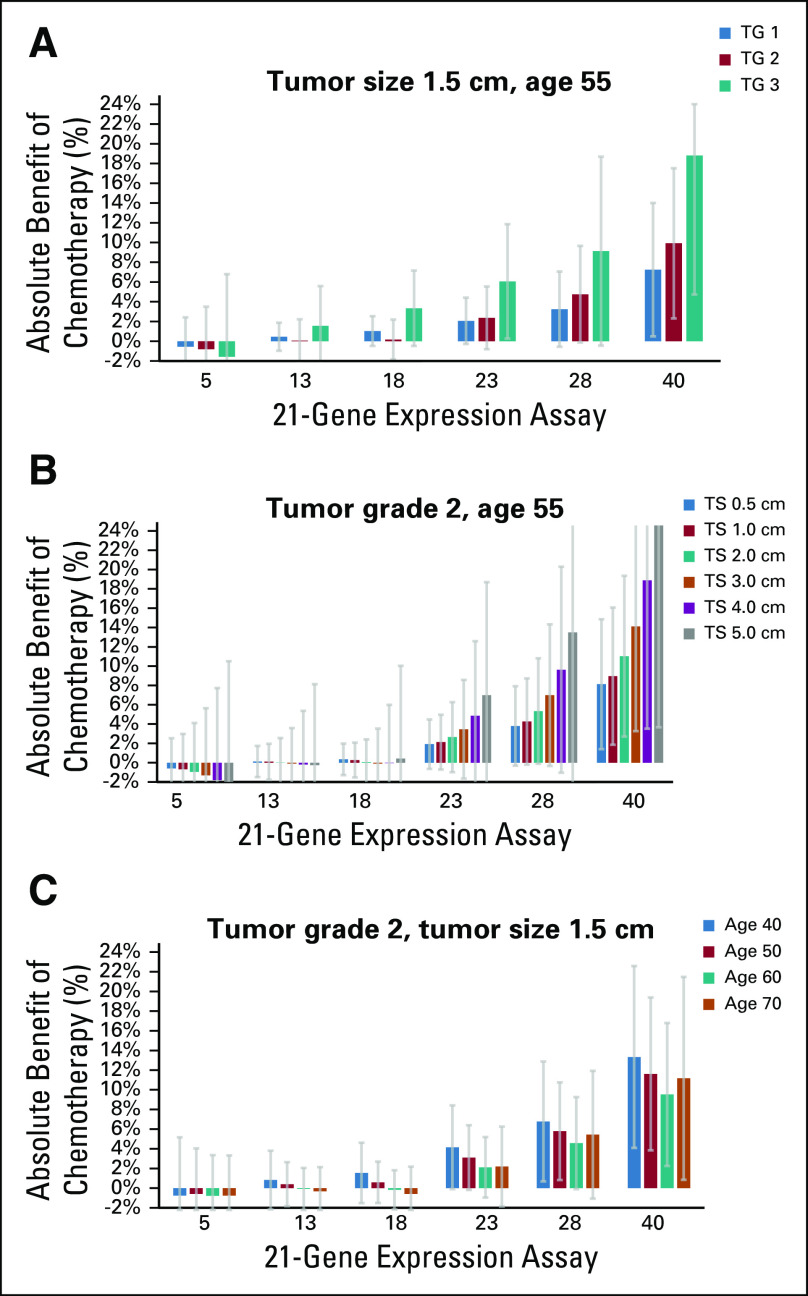
The RSClin tool provides individualized estimates for chemotherapy benefit based on the entry of patient information for the RS result, age, tumor size, and tumor grade. Example estimates and 95% CIs provided by the RSClin tool for the absolute benefit of adjuvant chemotherapy for (A) tumor grade series, (B) tumor size series, and (C) patient age series. RS, recurrence score.

Example estimates of absolute chemotherapy benefit are shown in Figure 2, demonstrating the impact of RS result, tumor grade, tumor size, and age on prognosis and the predictive effect of RS result on chemotherapy benefit. The results indicate that not all patients with unfavorable traditional risk factors have a large absolute chemotherapy benefit. Conversely, not all patients with favorable traditional risk factors have minimal or no absolute chemotherapy benefit.

**FIG 3.**
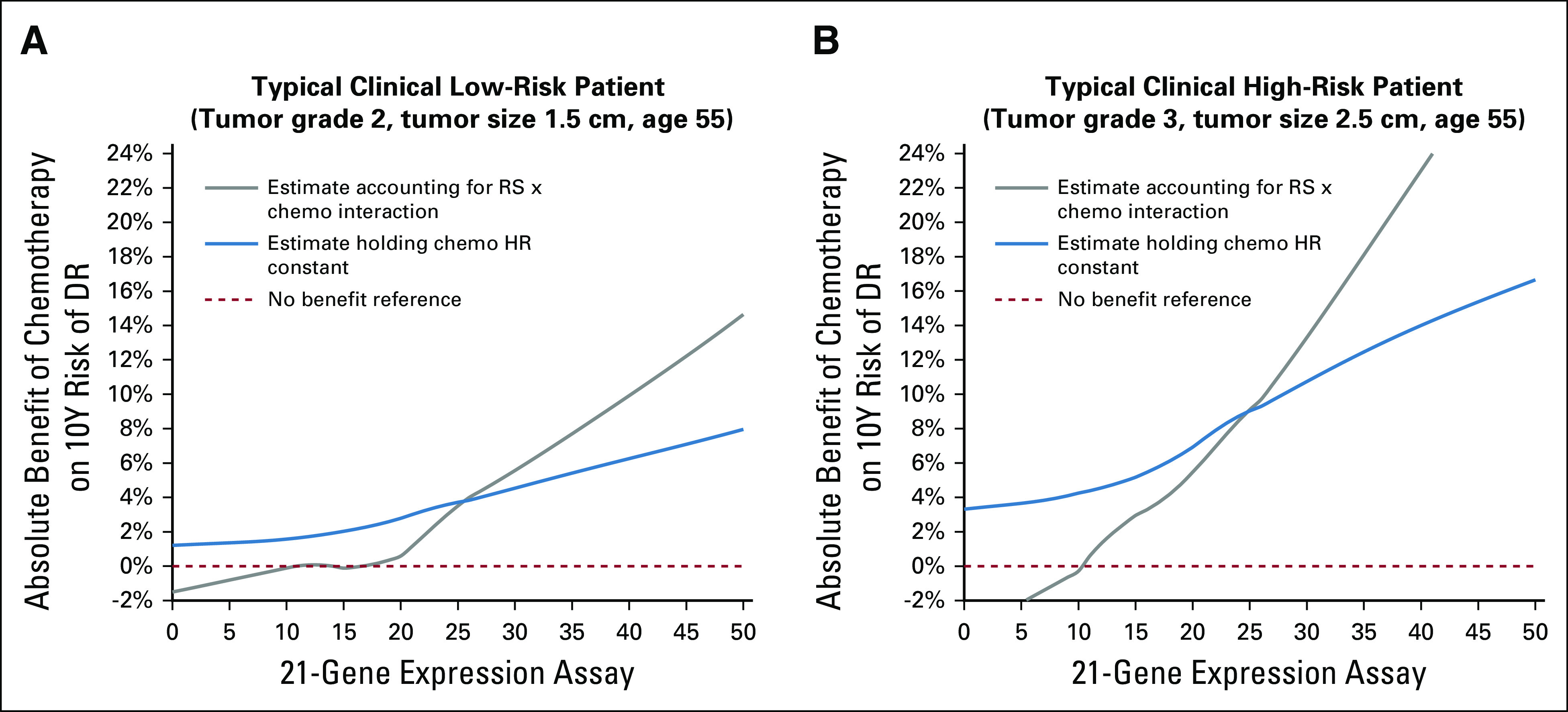
Comparison between estimate of absolute chemotherapy benefit provided by the RSClin tool (using PSMA estimate of relative chemotherapy benefit derived from B-20 and TAILORx) with estimate of absolute chemotherapy benefit if relative chemotherapy benefit was held constant (assuming higher RS result was associated with higher recurrence risk, but not greater relative chemotherapy benefit). Examples provided include (A) a 55-year-old woman with a 1.5-cm intermediate-grade tumor (a typical low clinical risk patient) and (B) a 55-year-old woman with a 2.5-cm high-grade tumor (a typically high clinical risk patient). RS, recurrence score.

To further illustrate the impact of prediction of chemotherapy effect by the 21-gene RS assay, Figure 3 shows two examples of absolute benefit estimates from the new prediction tool and the estimate where the relative benefit of chemotherapy was held constant, including typical examples of a clinical low-risk and clinical high-risk subject as defined in the TAILORx trial.^7^ Using the new prediction tool for a 55-year-old woman with a clinical low-risk 1.5-cm intermediate-grade tumor, the absolute chemotherapy benefit estimate ranges from 0% to 15% as the RS ranges from 11 to 50; this compares with a range of 2%-8% if the relative chemotherapy benefit did not vary by RS result and was held constant. Over the same RS range for a 55-year-old woman with clinical high-risk 2.5-cm poor-grade tumor, the tool's absolute chemotherapy benefit estimate ranges from 1% to 33% compared with 4% to 17% if the relative chemotherapy benefit was held constant. This indicates that incremental chemotherapy benefit observed with higher recurrence score is driven not only by a higher underlying recurrence risk but also by prediction of greater relative risk reduction with chemotherapy.

### External Validation of RSClin Risk Estimates in a Real-World Data Set

**FIG 4.**
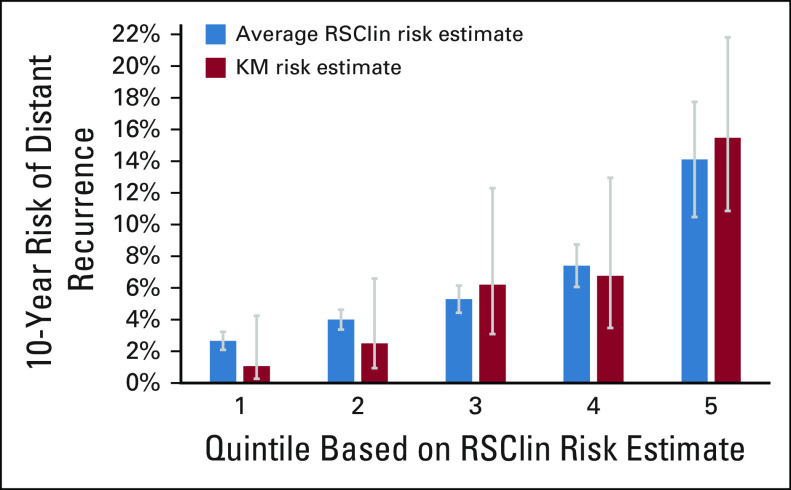
External validation of RSClin: Risk estimates provided by the RSClin tool versus real-world patient outcomes in the Clalit Health Services cohort: Analysis of the 1,098 patients divided into 5 quintiles based on increasing risk as calculated by the RSClin tool. Bars represent 95% CIs. The Lin concordance correlation is 0.962.

External validation was performed using data from 1,098 evaluable patients with node negative disease in the previously described Clalit Health Registry, of whom 876 received endocrine therapy alone and 222 received chemotherapy guided by the use of the 21-gene assay in addition to endocrine therapy (see Table S1 of the Data Supplement).^10^ The risk estimate provided by the RSClin tool was significantly associated with DR risk, with a standardized hazard ratio of 1.73 (95% CI, 1.40 to 2.15; *P* < .001). A calibration plot of the risk estimate provided by RSClin is shown in Figure 4. Within each quantile, the average estimated 10-year DR risk provided by the RSClin tool approximates the cohort Kaplan-Meier estimate, with a Lin concordance correlation of 0.962. As in the PSMA cohort, the RSClin tool provided more prognostic information than either RS result alone or clinical-pathological characteristics alone using likelihood ratio tests in patients who received endocrine therapy alone with linear effects for RS result, tumor size, and age (RSClin tool *v* PC [*P* = .027] and RSClin tool *v* RS result [*P* = .004]). Due in part to the strength of RS result in determining chemotherapy use in this cohort, propensity score matching could not appropriately be used for the evaluation of estimation of chemotherapy benefit by the RSClin tool (see page 76, Appendix S2 of the Data Supplement).

## DISCUSSION

In hormone receptor–positive, HER2-negative breast cancer, DR rates reflect the underlying recurrence risk, benefit from adjuvant endocrine therapy, and incremental benefit from adjuvant chemotherapy. The new RSClin prognostic tool integrates prognostic information provided by clinical-pathological features and the 21-gene RS assay in the context of contemporary adjuvant endocrine therapy and medical practice for estimation of 10-year DR rates in node-negative disease with endocrine therapy alone. It has also been enhanced to provide estimation of the absolute chemotherapy benefit expected in individual patients.

Comparison of the estimates of absolute chemotherapy benefit as a function of RS result provided by RSClin with estimates in which the relative chemotherapy benefit is held constant shows that RS result prediction contributes substantially to differentiation of treatment effect over and above the effect of increasing recurrence risk. Overall, the absolute magnitude of the chemotherapy benefit depends on the relative chemotherapy benefit associated with the individual's RS result and the individual's underlying DR risk that is influenced by both clinical-pathological features and the RS result.

The NSABP B-20 trial used cyclophosphamide, methotrexate, and fluorouracil (CMF) or MF. In TAILORx, of the patients with the RS result of 11-25 who received chemotherapy, 56% received taxane and cyclophosphamide, 36% an anthracycline-based regimen, and 7% CMF. RSClin estimates of absolute chemotherapy benefit use individualized chemotherapy hazard ratio estimates from a PSMA of TAILORx and B-20 for RS 11-25 and B-20 exclusively for RS outside this range. It is reassuring that the chemotherapy effects from TAILORx and B-20 are reasonably consistent (Data Supplement Fig S3). Nevertheless, the RSClin estimates of the absolute benefit of chemotherapy may be conservative, particularly for RS 26-100, relative to the most common current chemotherapy regimens.

Validation of the 21-gene RS assay in a HER2-negative subset of the NSABP B-20 cohort showed chemotherapy benefit in patients with an RS result of 26 or higher.^3^ The TAILORx trial found that there was no chemotherapy benefit in the overall TAILORx population with an RS result of 11-25, consistent with the previous finding that substantial chemotherapy benefit was obtained primarily in patients with higher recurrence score results. Subgroup analysis showed some chemotherapy benefit in women of age 50 or younger with RS results of 16-20 and 21-25 (1.6% and 6.5% absolute reduction in 9-year DR risk, respectively).^5^ Similar findings have been observed in a population-based cohort of 70,000 women with node-negative breast cancer.^15^ The new RSClin tool fills a need for more individualized estimation of absolute chemotherapy benefit.

It has been suggested that the absolute chemotherapy benefit in women of age 50 or younger with an RS result of 16-25 may be due to chemotherapy-induced early menopause.^7^ The use of ovarian function suppression plus an aromatase inhibitor rather than tamoxifen produces substantial reduction in recurrence risk comparable to chemotherapy^16^ and may be considered an option in circumstances where patients desire an alternative to chemotherapy.

Other decision aides provide prognostic information in early breast cancer.^17^ These prognostic models generally performed well in the development cohorts, with a relatively few validated in independent cohorts, especially in younger women. Two prognostic assays, EPclin and the Risk of Recurrence Score, incorporate clinical-pathological and genomic information.^18,19^ Advantages of the RSClin tool described in this report include the robust data set used in the meta-analysis, including younger women, use of DR rather than overall mortality as the end point, integration of predictive information for chemotherapy benefit provided by the RS result, and external validation in an independent cohort that reflects real-world clinical practice. Limitations include the lack of an external validation set including patients randomly assigned to chemotherapy.

In conclusion, we have developed a new prognostic tool called RSClin that reflects current medical practice for hormone receptor–positive, HER2-negative, and node-negative breast cancer, provides more accurate prognostic information than clinical-pathological features or RS result used individually, and externally validated the precision of the tool in providing prognostic estimates for 10-year DR rate in an independent real-world cohort. The RSClin tool has also been enhanced to now provide individualized estimates of absolute chemotherapy benefit. We have also shown that the individualized chemotherapy effect prediction provided by the RS result, based on contemporary treatments, contributes substantially to the estimate of absolute chemotherapy benefit. As demonstrated by this report, PSMA methods may be used in conjunction with randomized clinical trials to develop tools that facilitate more effective application of precision medicine in cancer therapy.

## Data Availability

Data from the NCI-sponsored trials (B14, B20, TAILORx) may be requested from the NCI NCTN Data Archive (https://nctn-data-archive.nci.nih.gov). Data from the CLALIT registry may be requested from the study chair (SMS). Data requests may also be submitted to the corresponding author, who may assist in coordinating the request from the appropriate sources.
